# Orofacial pain and oral health-related quality of life in woodwind and cello musicians in German orchestras: an online based questionnaire study

**DOI:** 10.1186/s12995-025-00467-4

**Published:** 2025-06-06

**Authors:** Felix Marschner, Armin Sokolowski, Alwin Sokolowski, Jana Biermann, Annette Wiegand

**Affiliations:** 1 Department of Preventive Dentistry, Periodontology and Cariology, University Medical Center Göttingen, Robert-Koch-Str. 40, Göttingen, 37075 Germany; 2 Divison of Restorative Dentistry, Periodontology and Prosthodontics, Department of Dental Medicine and Oral Health, Medical University of Graz, Billrothgasse 4, Graz, 8010 Austria

**Keywords:** Musician, Orchestra, Orofacial pain, Quality of life, Stress, Sleep disorder

## Abstract

**Background:**

Occupational factors and the type of instrument played may influence physical and psychological health, affecting oral health-related quality of life (OHRQoL). This study assessed the prevalence of orofacial pain, sleep bruxism, stress, and OHRQoL among woodwind musicians (oboe, flute, clarinet, bassoon) in German professional orchestras, compared to cellists.

**Methods:**

A standard online questionnaire was sent to all 129 German professional orchestras. Orofacial pain, stress, and sleep-related issues in the past 30 days were evaluated. The German version of the Oral Health Impact Profile-14 (OHIP-14) assessed OHRQoL. Logistic and linear regression analyses were performed (statistical significance *p* < 0.05).

**Results:**

A total of 243 musicians were included. Orofacial pain was reported by 35.8%, sleep bruxism by 63.0%, and stress by 88.9% of the participant. Orofacial pain was significantly associated with female gender (*p* = 0.027; odds ratio [OR] = 2.09, 95% confidence interval [CI]: 1.09–4.02), frequent sleep bruxism (*p* = 0.013; OR = 2.65, 95%-CI: 1.23–5.69), frequent stress (*p* = 0.002; OR = 3.19, 95%-CI: 1.53–6.63), and difficulties initiating sleep after evening shifts (*p* = 0.003; OR = 2.90, 95%-CI: 1.45–5.80), but not with the instrument played. OHIP-14 scores did not differ significantly between instrument groups (*p* = 0.629), but correlated with orofacial pain (*p* < 0.001), sleep bruxism (*p* < 0.001), stress (*p* = 0.002), and sleep difficulties (*p* = 0.040).

**Conclusions:**

Orofacial pain and sleep bruxism are common among professional musicians, with stress-related factors playing a more significant role than the instrument played.

**Trial registration:**

ClinicalTrials.gov, NCT06618898, 27.09.2024.

## Background

Germany has a rich cultural landscape, characterized by historically developed structures with internationally significant orchestras. Considering its population size, Germany has the highest density of professional orchestras in the world (129 professional orchestras) [1].

Professional musicians in these orchestras are exposed to a variety of occupational factors that can potentially be harmful to their health [2–5]. These factors include environmental noise, irregular working hours, tight schedules, and high demands for quality and discipline [2, 6]. These factors can cause stress, sleep problems, and physical impairments [7].

Besides the work environment, the type of instrument played may be related to physical and psychological problems that could affect musicians’ occupational performance and further increase their stress experience [8]. Stress and insufficient sleep are associated with sleep bruxism [9, 10]. Bruxism is defined as repetitive jaw muscle activity, characterized by teeth clenching, grinding, or by bracing or thrusting of the mandible and has been identified as a possible cause of orofacial pain [11]. Additionally, sleep bruxism is considered in general as a potential risk factor for temporomandibular disorders [12].

So far, Ahlberg et al. [6] found an association between orofacial pain, stress experience, sleep bruxism, and disrupted sleep among musicians in symphony orchestras. While the majority of studies have focused on professional musicians in general [6, 10, 13], studies focusing on specific musicians as a potentially vulnerable group are lacking so far. Woodwind instruments, such as the oboe, flute, clarinet, and bassoon, produce sound through the vibration of air, either by a reed or a blowing technique. Woodwind musicians are exposed to orofacial pain and symptoms of temporomandibular disorders due to their playing techniques, which affect musculoskeletal function by overloading their masticatory and facial muscles [14–17]. In this context, limitations in oral health-related quality of life (OHRQoL) are conceivable.

Hence, this prospective online questionnaire-based study aimed to examine the association between the type of woodwind instrument played (oboe, flute, clarinet, and bassoon), orofacial pain, sleep-related issues, and OHRQoL among professional musicians, compared to a control group of cellists in German orchestras. Professional cellists served as control, as their playing technique may have less impact on the orofacial system, while work-related factors remain comparable to those of woodwind musicians in professional orchestras [6].

## Methods

The present study was performed between November 2024 and January 2025 and followed the guidelines of the Declaration of Helsinki. The research protocol was approved by the ethics committee of the Medical Center Göttingen (13/9/24) and was registered at ClinicalTrials.gov (NCT06618898).

### Data collection

In November 2024, a standard online questionnaire was sent via email to the orchestral offices of all professional orchestras in Germany (*n* = 129) to address woodwind (oboe, flute, clarinet, and bassoon) and cello musicians. Additionally, the German Orchestra Union (unisono, Berlin, Germany), which serves as the professional association and labor union for orchestra musicians, distributed the online questionnaire to all its members, inviting them to participate in the study. After six weeks, a reminder was sent via email to the orchestral offices and through the newsletter to unisono members. The survey was conducted using a web-based survey platform (www.evasys.de). The musicians were informed about the aim of the study and the anonymous nature of the survey. Informed consent was implied through voluntary completion of the questionnaire.

Inclusion criteria were:

- Age: ≥ 18 years
- Musicians whose primary instruments are oboe, flute, clarinet, bassoon, or cello
- At least 12 months of employment or freelance work in the past 5 years with a German professional orchestra

Exclusion criteria was:

- Missing consent

### Online questionnaire

The online questionnaire was modified from Ahlberg et al. [6] and included the following demographic data: age, gender, instrument group (oboe, flute, clarinet, bassoon, or cello), years of service, number of services in the last six months and specific questions about orofacial pain, sleep bruxism, stress experiences, and sleep quality [18].

The following specific questions were used:

- Orofacial pain: During the past 30 days, how long did any pain last in your jaw or temple area on either side? (no pain, pain is present occasionally, pain is continuous).
- Sleep bruxism: Sleep bruxism is involuntary periodical tooth grinding or tooth clenching. Do you have such symptoms? (never, seldom, occasionally, almost every night, every night).
- Orofacial pain treatment history: Have you ever received medical, dental, or physiotherapeutic treatment for jaw and facial pain or teeth grinding? (yes, no).
- Stress experience: Stress means the situation when a person feels tense, restless, nervous or anxious, or is unable to sleep because his/her mind is troubled. Do you feel that kind of stress these days? (not at all, seldom, to some extent, rather much, very much).
- Difficulties in initiating sleep after an evening shift: How long does it usually take you to fall asleep? (< 10 min, 10–30 min, > 30 min).
- Difficulties in initiating sleep on days without an evening shift: How long does it usually take you to fall asleep? (< 10 min, 10–30 min, > 30 min).
- Sleep interruptions after an evening shift: How often do you usually wake up during the night? (0-1 times, 2‐3 times, 4 times or more).
- Sleep interruptions on days without an evening shift: How often do you usually wake up during the night? (0-1 times, 2‐3 times, 4 times or more).
- Non-restorative sleep: How often do you feel refreshed after awakening? (never or once a week, 2‐4 times a week, 5 or more times a week).
- Tiredness: How often do you feel tired or non-energetic during daytime? (never or once a week, 2‐4 times a week, 5 or more times a week).

Moreover, the German version of the Oral Health Impact Profile-14 (OHIP-14) questionnaire was used to assess the OHRQoL of the participants [19, 20]. The OHIP-14 investigates the impact of oral conditions on oral health-related well-being, including following domains: functional limitation, physical pain, psychological discomfort, physical disability, psychological disability, social disability, and handicap [19]. This validated questionnaire consists of 14 questions [21]. All items were graded on a 0–4 scale: never = 0, hardly ever = 1, sometimes = 2, quite often = 3, and very often = 4 [19]. The total score is calculated by adding up the points from the 14 items, with the OHIP-14 score ranging from 0 to 56. Higher scores indicate a lower OHRQoL [21].

### Statistical methods

Descriptive data were summarized as means (± standard deviation) or percentages. Differences in continuous variables between instrument groups were assessed using Kruskal-Wallis-test, with post-hoc Dunn’s test and Bonferroni correction for multiple comparisons. Categorical variables were analyzed using chi-square test. Logistic regression model was applied to analyze the probability of current orofacial pain (any pain = 1, no pain = 0) with the following variables: Instrument groups (woodwinds = 1, cello = 0), sleep bruxism (almost every night or every night = 1, else = 0), stress experience (rather much or very much = 1, else = 0), difficulties in initiating sleep after an evening shift and on days without an evening shift (30 min or more = 1, else = 0), sleep interruptions after an evening shift and on days without an evening shift (4 times or more = 1, else = 0), and tiredness (5 or more times a week = 1, else = 0). The relationship between OHIP-14 scores and variables was analyzed using linear regression. The OHIP-14 scores were logarithmically transformed due to the non-normal distribution of the data. Model performance was evaluated using Nagelkerke R² and McFadden R² for logistic regression, and R² and adjusted R² for linear regression. The level of significance was set to p-value < 0.05. Statistical analysis was performed using JASP (Version 0.19.3).

## Results

A total of 249 musicians completed the standard online questionnaire, six participants did not meet the eligibility criteria, and were excluded. The characteristics of the study population (*N* = 243) according to the instrument groups are reported in Table 1.

The mean age of musicians was 45.62 ± 12.5 years, flutists were significantly older than clarinetists (p_adj_. = 0.020). 51.0% of musicians were women. The proportion of women was significantly higher among flutists compared to bassoonists (p_adj_. = 0.011), clarinetists (p_adj_. = 0.032), and cellists (p_adj_. = 0.008). The mean years of service were 20.5 ± 12.6 years. Flutists had significantly more years of service than clarinetists (p_adj_. = 0.018). All musicians reported a high level of services in the past six months (110.3 ± 41.7). More than one-third of musicians (35.8%) reported orofacial pain, while sleep bruxism, stress experience, and a history of orofacial pain treatment were reported by 63.0%, 88.9%, and 31.7%, respectively, with no statistical significance between instrument groups (*p* > 0.05). Regarding sleep parameters only sleep interruptions on days without an evening shift was statistically significant between the instrument groups (*p* = 0.024).

**Fig. 1:**
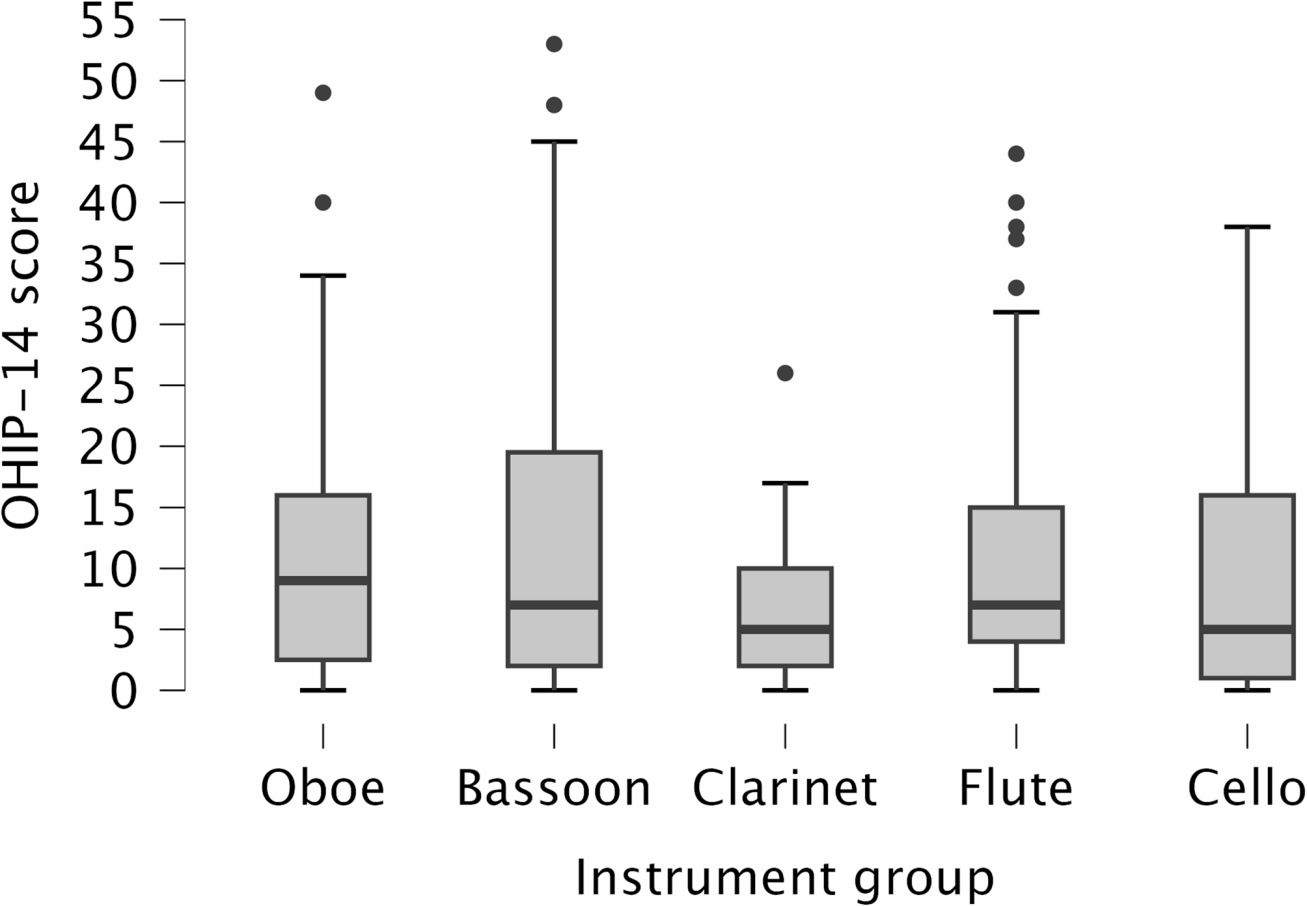
Mean OHIP-14 scores for different instrument groups. Boxes represent median with interquartile range and whiskers represent the 5th-95th percentile. Outliers (> Q3 + 1.5*IQR) are marked with a dot (•)

**Fig. 2:**
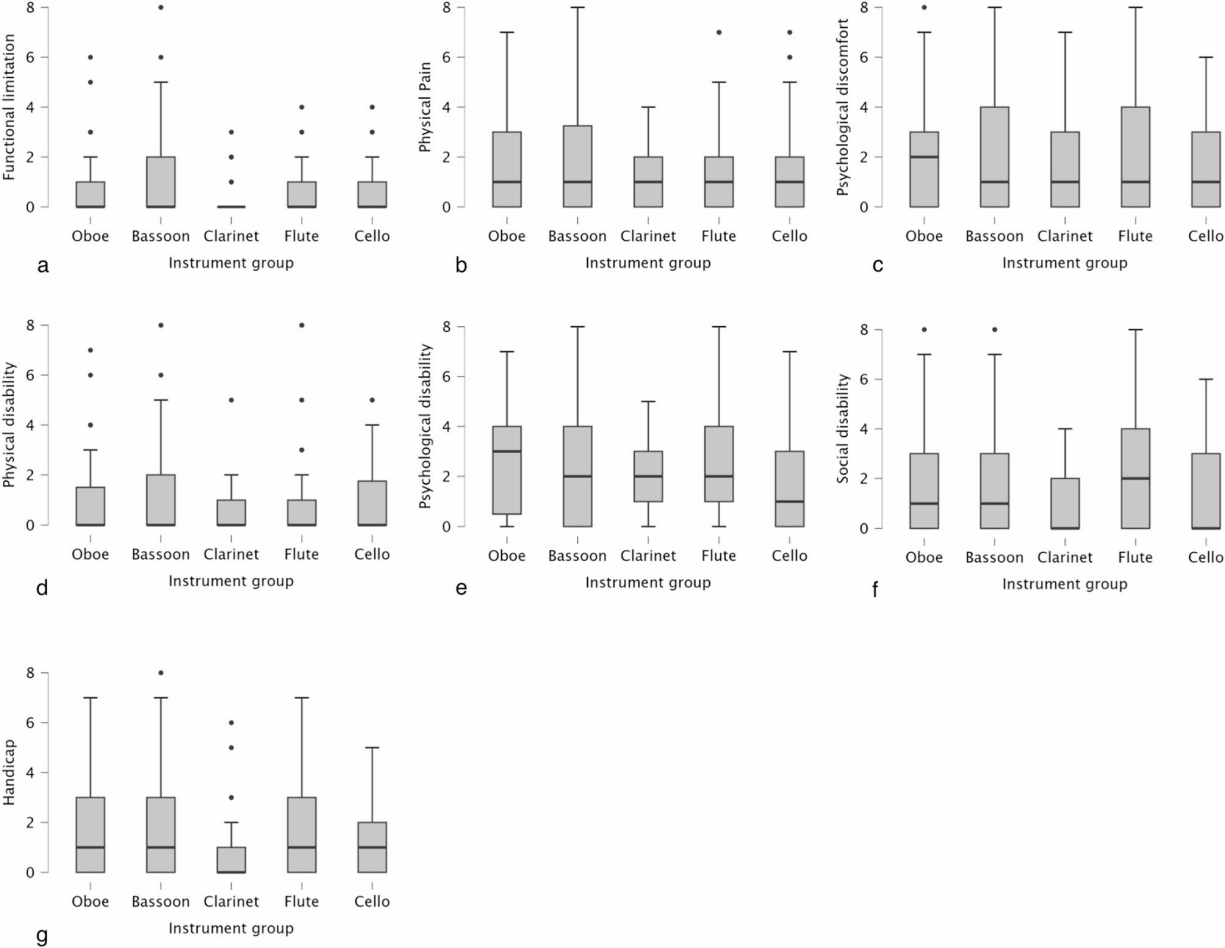
Mean scores for OHIP-14 domains for different instrument groups. **a** functional limitation, **b** physical pain, **c** psychological discomfort, **d** physical disability, **e** psychological disability, **f** social disability, **g** handicap, boxes represent median with interquartile range and whiskers represent the 5th-95th percentile. Outliers (> Q3 + 1.5*IQR) are marked with a dot (•)

The mean OHIP-14 score among all musicians was 10.2 ± 10.9. Figure 1 shows the different OHIP-14 score between the instrument groups. No significant differences were found between the instrument groups (*p* = 0.629). Similarly, no significant differences were observed among the OHIP-14 domains (*p* > 0.05). Scores for OHIP-14 domains are shown in Fig. 2a-g.

Logistic regression revealed that orofacial pain was significantly associated with female gender (*p* = 0.027), frequent sleep bruxism (*p* = 0.013), frequent stress experience (*p* = 0.002), and difficulties in initiating sleep after an evening shift (*p* = 0.003). Table 2 presents the odds ratios with their 95% confidence interval for each included variable.

**Table 1:** Descriptive data on the study population according to the instrument groups

	Instrument group					
Total (*N* = 243)	Oboe (*n* = 43)	Bassoon (*n* = 44)	Clarinet (*n* = 49)	Flute (*n* = 45)	Cello (*n* = 62)	*p*-value
Age in years (mean ± SD)	44.0 ± 12.3	47.7 ± 13.3	41.0 ± 13.2	49.4 ± 11.0	46.1 ± 11.5	0.022*
Gender (%)						< 0.001*
Male	34.9	61.4	57.1	26.7	59.7	
Female	65.1	38.6	42.9	73.3	40.3	
Years of service (mean ± SD)	18.8 ± 11.9 (*n* = 42)	23.1 ± 12.7 (*n* = 43)	16.1 ± 13.9 (*n* = 49)	24.2 ± 11.3 (*n* = 45)	20.5 ± 11.9 (*n* = 61)	0.014*
Services in the last six months (mean ± SD)	99.7 ± 32.4 (*n* = 37)	102.1 ± 34.6 (*n* = 40)	121.3 ± 53.6 (*n* = 43)	105.2 ± 40.2 (*n* = 43)	118.8 ± 40.3 (*n* = 54)	0.059
Orofacial pain (%)						0.117
No pain	62.8	70.5	59.2	60.0	67.7	
Occasionally	30.2	15.9	40.8	28.9	25.8	
Continuous	7.0	13.6	0.0	11.1	0.0	
Sleep bruxism (%)						0.803
Never	27.9	36.4	40.8	35.6	41.9	
Seldom	23.3	22.7	16.3	11.1	24.2	
Occasionally	25.6	18.2	16.3	26.7	16.1	
Almost every night	14.0	11.4	18.4	15.6	6.5	
Every night	9.3	11.4	8.2	11.1	11.3	
Orofacial pain treatment history (%)						0.208
Yes	32.6	29.5	32.7	44.4	22.6	
No	67.4	70.5	67.3	55.6	77.4	
Stress experience (%)						0.204
Not at all	9.3	6.8	12.2	13.3	12.9	
Seldom	20.9	18.2	30.6	22.2	29.0	
To some extent	41.9	40.9	32.7	26.7	24.2	
Rather much	20.9	15.9	24.5	22.2	27.4	
Very much	7.0	18.2	0.0	15.6	6.5	
Difficulties in initiating sleep after an evening shift (%)						0.809
< 10 min	25.6	18.2	32.7	24.4	22.6	
10–30 min	27.9	36.4	28.6	37.8	29.0	
> 30 min	46.5	45.5	38.8	37.8	48.4	
Difficulties in initiating sleep on days without an evening shift (%)						0.325
< 10 min	58.1	40.9	40.8	53.3	43.5	
10–30 min	27.9	52.3	53.1	40.0	45.2	
> 30 min	14.0	6.8	6.1	6.7	11.3	
Sleep interruptions on days without an evening shift (%)						0.024*
0-1 times	90.7	65.9	71.4	77.8	59.7	
2-3 times	9.3	31.8	28.6	22.2	35.5	
4 times or more	0.0	2.3	0.0	0.0	4.8	
Restorative sleep (%)						0.923
Never or once a week	23.3	18.2	14.3	22.2	21.0	
2-4 times a week	53.5	56.8	57.1	44.4	53.2	
5 or more times a week	23.3	25.0	28.6	33.3	25.8	
Tiredness (%)						0.873
Never or once a week	25.6	25.0	28.6	28.9	25.8	
2-4 times a week	65.1	56.8	65.3	60.0	62.9	
5 or more times a week	9.3	18.2	6.1	11.1	11.3	

Kruskal-Wallis-test for the group means; chi-square test for categorical variables; *statistical significance (*p* < 0.05); Abbreviation: *SD* Standard deviation

**Table 2:** Relationships between orofacial pain and variables

Variables	OR	95% CI	*p*-value
Age (years)	0.95	0.93–0.98	< 0.001*
Gender (female)	2.09	1.09–4.02	0.027*
Instrument (woodwind)	1.34	0.62–2.88	0.457
Sleep bruxism (almost every night or every night)	2.65	1.23–5.69	0.013*
Stress experience (rather much or very much)	3.19	1.53–6.63	0.002*
Difficulties in initiating sleep after an evening shift (30 min or more)	2.90	1.45–5.80	0.003*
Difficulties in initiating sleep on days without an evening shift (30 min or more)	0.40	0.13–1.26	0.118
Sleep interruptions after an evening shift (4 times or more)	2.56	0.69–9.53	0.162
Sleep interruptions on days without an evening shift (4 times or more)	1.00	0.10-10.56	0.998
Tiredness (5 or more times a week)	0.42	0.15–1.16	0.094

Logistic regression, Nagelkerke R² = 0.37, McFadden R² = 0.24; *, statistical significance (*p* < 0.05); *Abbreviation*: *OR* Odds ratio, *CI* Confidence interval

The results of linear regression for the relationship between OHIP-14 score and included variables are shown in Table 3. The presence of orofacial pain (*p* < 0.001), sleep bruxism (*p* < 0.001), stress experience (*p* = 0.002), and difficulties in initiating sleep after an evening shift (*p* = 0.040) were significantly associated with higher OHIP-14 scores and consequently with a lower OHRQoL.

**Table 3:** Relationships between OHIP-14 score and variables

Variables	Exp(B)	Exp (95% CI)	*p*-value
Age (years)	1.00	0.99–1.01	0.624
Gender (female)	1.07	0.84–1.38	0.563
Instrument (woodwind)	1.12	0.85–1.46	0.427
Orofacial pain (pain)	1.92	1.45–2.56	< 0.001*
Sleep bruxism (almost every night or every night)	1.80	1.31–2.48	< 0.001*
Stress experience (rather much or very much)	1.60	1.19–2.16	0.002*
Difficulties in initiating sleep after an evening shift (30 min or more)	1.34	1.01–1.75	0.040*
Difficulties in initiating sleep on days without an evening shift (30 min or more)	0.81	0.52–1.26	0.351
Sleep interruptions after an evening shift (4 times or more)	1.17	0.71–1.95	0.525
Sleep interruptions on days without an evening shift (4 times or more)	0.72	0.26–1.93	0.507
Tiredness (5 or more times a week)	1.30	0.87–1.93	0.195

Linear regression, R² = 0.36, adjusted R² = 0.33; *, statistical significance (*p* < 0.05); *Abbreviation*: *Exp(B)* exponentiated regression coefficient, *Exp(CI)* Exponentiated confidence interval

## Discussion

The aim of this study was to evaluate the association between the type of woodwind instrument (oboe, flute, clarinet, and bassoon) played, orofacial pain, sleep bruxism, stress experience, sleep-related issues, and OHRQoL among professional musicians in German orchestras, compared to a control group of cellists. Orofacial pain is a common complaint among all included professional musicians, with a self-reported prevalence of 35.8%. This finding is in accordance with a previous study indicating that a significant proportion of musicians (28.9%) frequently experience orofacial pain [6]. It has been suggested that musicians who play woodwind instruments may place excessive strain on their masticatory and facial muscles to maintain control while playing [15, 16, 22, 23]. Surprisingly, we did not find significant differences in orofacial pain prevalence between woodwind musicians and cellists. Previous studies have confirmed that factors beyond playing technique contribute to the development of orofacial pain [6, 24, 25]. Additionally, stratifying the data by gender showed no significant differences between woodwind musicians and cellists within either gender.

Sleep bruxism was reported by 63.0% of the participants, a high prevalence compared to general population estimates [26]. Regarding the well-established association between stress and sleep bruxism [27], it is not surprising that stress levels in our participants were also high (88.9%). Professional orchestral work, characterized by irregular schedules, performance pressure, and intense competition, likely contributes to this elevated stress burden [14, 28]. Our findings support previous studies that have identified professional musicians as a high-risk group for stress-related conditions [6, 29, 30].

Logistic regression revealed that orofacial pain was significantly associated with female gender, frequent sleep bruxism, frequent stress, and difficulties initiating sleep after evening shifts. The association between female gender and orofacial pain is consistent with previous studies indicating a higher prevalence of temporomandibular disorders and orofacial pain among women [31, 32]. Hormonal factors, differences in pain perception, and coping mechanisms may contribute to this disparity [32]. Structural factors such as the underrepresentation of women in leadership positions within orchestras and experiences of workplace discrimination, and harassment may contribute to the higher prevalence of stress-related complaints among female musicians. In German professional orchestras, only about 28.4% of principal positions are held by women [33]. Moreover, more than two-fifths of female musicians report having experienced workplace harassment [34]. These factors may increase stress levels and the risk of orofacial pain and should be considered in future research [35].

Moreover, frequent sleep bruxism emerged as a significant predictor of orofacial pain, supporting the hypothesis that repetitive jaw muscle activity contributes to musculoskeletal overload [36]. Frequent stress experience was another factor associated to self-reported orofacial pain. Stress has been shown to increase muscle tension and exacerbate pain conditions in the craniofacial region [37, 38]. Additionally, difficulties initiating sleep after evening shifts were associated with orofacial pain, suggesting that disrupted sleep patterns and inadequate recovery time may exacerbate musculoskeletal burden [39].

Beside the high prevalence of orofacial pain, sleep bruxism, and stress, our findings did not find significant differences in mean OHIP-14 scores between instrument groups. However, we observed significant association between OHIP-14 scores and orofacial pain, sleep bruxism, stress, and sleep difficulties, indicating that these factors are associated with lower OHRQoL. Interestingly, the mean OHIP-14 scores of musicians were above the median values of the general population in Germany [40].This finding underscores the functional and psychosocial burden of orofacial pain in musicians, potentially affecting their professional performance and OHRQoL. Furthermore, sleep bruxism, stress, and difficulties in initiating sleep on days without an evening shift were linked to lower OHRQoL.

The findings of the present study emphasize the importance of developing preventive strategies to address specific health challenges faced by professional musicians in German orchestras. It is crucial for musicians, healthcare professionals, and orchestra management to be aware of the high prevalence of stress-related conditions, and sleep disturbances, which may also result from late-night work typical in orchestral settings. A key limitation of our study was in the use of anonymous, self-reported data, which may be prone to recall bias, particularly regarding sleep bruxism and stress experiences. This limitation is attributed to the exploratory nature of this pilot study, which, however, provides the first data in this research field, and is also influenced by the nature of employment in the musical profession.

## Conclusion

Future prospective clinical studies with detailed clinical assessments (e.g., with standardized pain scales) are necessary for a better understanding of orofacial pain, physical and psychological challenges faced by high-performing groups, such as orchestra musicians. These studies should include larger and more diverse samples across different instrument groups, age ranges, and levels of professional experience.

## Data Availability

All data generated or analyzed during this study are included in this published article.

## Abbreviations

CI: Confidence interval
Exp(B): Exponentiated regression coefficient
Exp(CI): Exponentiated confidence interval
OHIP-14: Oral Health Impact Profile-14
OHRQoL: Oral health-related quality of life
OR: Odds ratio
SD: Standard deviation
